# Carbon nanotube/Chitosan hydrogel for adsorption of acid red 73 in aqueous and soil environments

**DOI:** 10.1186/s13065-023-01019-9

**Published:** 2023-08-24

**Authors:** Jia Wei, Luchun Yan, Zhifang Zhang, Bing Hu, Wenjun Gui, Yanjun Cui

**Affiliations:** 1 College of Science, Gansu Agricultural University, Lanzhou, Gansu, 730070 China; 2 Gansu Henglu Traffic Survey and Design Institute, Lanzhou, Gansu, 730070 China

**Keywords:** Pollution treatment, AR73 pollutant, Environment-friendly material, Recyclable

## Abstract

Acid red 73 is an azo dye, and its residue can pollute the environment and seriously threaten human health and life. In this study, glutaraldehyde was used as the crosslinking agent, chitosan and polyvinyl alcohol were crosslinked under appropriate conditions to obtain a chitosan hydrogel film, and carbon nanotubes were dispersed in the chitosan hydrogel film. The FTIR, XRD, BET, SEM were applied to chatacterize the structure and the morphology of the absorbent and results showed that when the mass fraction of the carbon nanotubes was 1%, the structure was a three-dimensional network with microporous, and the water absorption reached to the maximum value of 266.07% and the elongation at break reached to a maximum of 98.87%. The ability to remove acid red 73 from aqueous and soil environments was evaluated by UV. In the aqueous samples, 70 mg of the adsorbent reached a saturated adsorption capacity of 101.07 mg/g and a removal rate of 92.23% at pH = 5. The thermodynamics conformed with the Langmuir adsorption isotherm and pseudo second-order adsorption kinetic models. In the soil samples, 100 mg of the adsorbent reached an adsorption capacity of 24.73 mg/g and removal rate of 49.45%. When the pH of the soil is between 4 and 7, the removal rate and adsorption capacity do not change much; hence, the pH should be maintained between 5.2 and 6.8, which is extremely suitable for the growth of general plants. Moreover, the experimental results demonstrated that the adsorbent maintained a good removal rate of acid red 73 over six adsorption cycles.

**Graphical Abstract:**

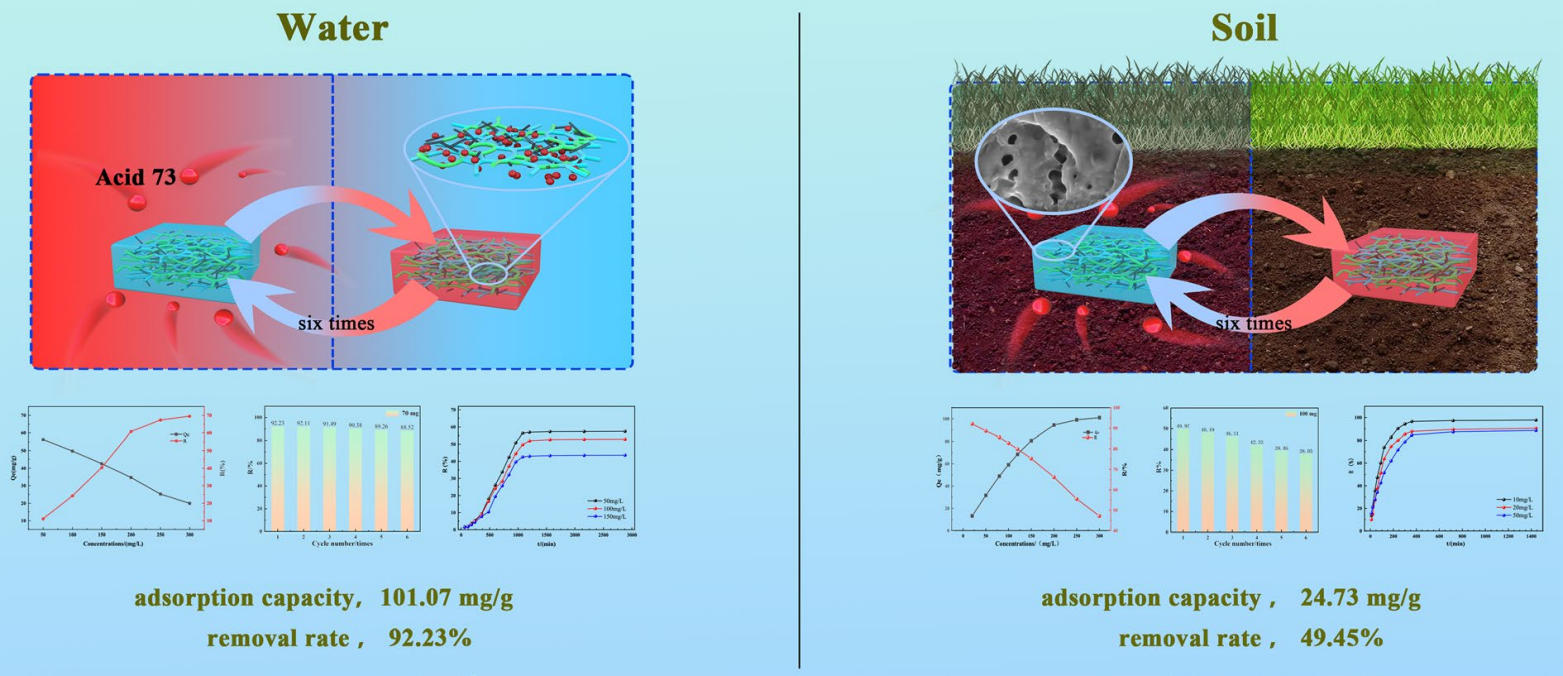

**Supplementary Information:**

The online version contains supplementary material available at 10.1186/s13065-023-01019-9.

## Introduction

Industrial development has resulted in increasing contamination of aqueous and soil environments by various types of pollution [1–4]. Dyes can be hazardous to the environment and human health due to their toxicity and biodegradability [5–8]. Azo dyes comprise azo groups, which are insoluble organic compounds, and they are widely applied in different industries such as pharmaceuticals, cement, and paper [9, 10]. Around the world, almost 500,000 T of azo dyes is discharged into water bodies each year, which has caused serious pollution of aqueous and soil environments. Acid red 73 (AR73) is a typical azo dye with high toxicity. Accumulated AR73 in the environment can enter the human body, which can cause cancer when exposed for a prolonged period [11, 12]. AR73 has stable N = N groups, benzene rings, and a naphthalene ring, which limit its purification by ordinary biological methods. Therefore, the residue of AR73 needs to be decolorized. Research on dye decolorization has attracted wide attention [13–15]. Decolorization efficiently removes pollutants from wastewater via physical and chemical adsorption at a low cost without causing secondary pollution [16]. Using appropriate adsorbent material improves the pollutant absorption [17, 18].

Due to the increasing development of industries, several types of environmental pollution have emerged, which seriously threaten the environment and human safety. The pollutants in the environment include organic pollutants, heavy metal pollutants, and biological pollutants, among others. Among the organic pollutants, dyes are one of the important pollution sources. At present, methods exist for treating dye pollutants, including the adsorption method, precipitation method, biological treatment method, ion exchange method, and membrane separation method. Each method has its own advantages and disadvantages, among which the adsorption method has the advantages of simple operation, low cost, and large adsorption capacity; thus, it is widely used and has attracted the attention of researchers [19–21].

Three-dimensional porous network adsorbents are widely used owing to their high specific surface area, low density, and reproducibility [22–24]. Praveen et al. [25] demonstrated that coconut–shell biochar can remove basic red 09 from wastewater at a maximum adsorption capacity of 44 mg/g. Hidayat et al. [26] prepared a chitosan adsorbent crosslinked with zeolite (ZL–CH) to extract acid red 88 (AR88) from wastewater. ZL–CH exhibited an adsorption capacity of 332.48 mg/g (equilibrium time = 1 min), and NaOH acted as the desorbing agent with 93.8% efficiency. The adsorption kinetics was also studied, and the results agree well with the pseudo second-order kinetic model. Therefore, ZL–CH is a promising AR88 adsorbent. TGonçalves et al. [27] developed an adsorbent by modifying a chitosan hydrogel scaffold with carbon nanotubes (CNTs), which were used to remove the food dyes red 17 (FdR17) and blue 1 (FdB1) from single and binary solutions. For FdR17, its adsorbent reached maximum adsorption capacities of 1508 and 955 mg/g in the single and binary solutions,, respectively. For FdB1, its adsorbent exhibited maximum adsorption capacities of 1480 and 902 mg/g in the single and binary solutions, respectively. Wang et al. [28] developed an adsorbent from rice to remove AR73 from an aqueous solution, and the adsorption capacity remained at 71% of the initial value after four cycles. The Freundlich equation accurately described the adsorption isotherm, and the maximum adsorption capacity was 18.74 mg/g.

The above studies mostly focused on treating azo dye-contaminated water. Studies have considered the adsorption of AR73 in aqueous solutions with different adsorption capacities mostly under 40 mg/g [29–32].Soil environments are more complex than aqueous solutions, and treating contaminated soil with adsorbents in the form of powder or microspheres can lead to secondary pollution because such adsorbents are difficult to recover. Therefore, only a few studies have focused on treating azo dye-contaminated soil so far.

Chitosan is a naturally occurring linear polymer, which is sufficiently available in nature. It is the only linear alkaline amino polysaccharide and only natural positive polyelectrolyte among natural polysaccharides. As a treatment ansorbent for environmental pollutants, chitosan has the advantages of multiple high-abundance sources and environmental friendliness. Previous experimental studies have indicated that chitosan hydrogel is an attractive adsorbent material for azo dyes. Moreover, previous studies has shown that pure chitosan hydrogels can be modified to improve their properties and adsorption performance [33–37].

To effectively remove acid red 73 pollutants from water and soil simultaneously, this study prepared carbon nanotubes/chitosan hydrogel film adsorption materials, determined the best preparation conditions for the materials, and studied the removal ability of adsorption materials in water and soil acid red 73 pollution under different influencing factors [38–40].

Chitosan was selected as the base substrate to prepare materials, and a high-strength chitosan-based interpenetrating network hydrogel film was formed with added nonionic, hydrophilic, and polyvinyl alcohol polymer, with glutaraldehyde as the crosslinking agent with an excellent absorbtion property [41–43]. Li [42] et al. developed a chitosan modified by MnFe_2_O_4_ nanoparticlesan to remove As(III)), Cd(II), Cu(II), and Pb(II) with maximum adsorption capacities of 9.90, 9.73, 43.94, and 11.98 mg/g, respectively within 8 h. Chitosan hydrogel molecules contain a large number of –NH_2_ functional groups that can be ionized into –NH_3_^+^ groups in water, further enhancing its swelling, water absorption capacities, and mechanical properties,which can be further improved by adding multiwalled carbon nanotubes (MWCNTs) into the hydrogels.

The findings of this study demonstrate that the incorporation of 1-wt% MWCNTs increased the absorption capacity of the carbon nanotube/chitosan hydrogel in water to a much higher value than that reported in the existing literature. In this study, when the mass of adsorbent was more than 70 mg, the initial concentration of AR73 was 10 mg/L, and the mass concentration of AR73 was less than 1.4 mg/L after 3 h of adsorption, which was in line with the GB-4287-2102 National Standard.The absorption capacity of this absorbent was also studied in soil, demonstrating performance better than some in-water systems. Additionally, the carbon nanotube/chitosan hydrogel film in this study could maintain the basic absorption ability even after six cycles of usage.

### Hypothesis

Modifying a chitosan hydrogel with multiwalled carbon nanotubes will realize a composite adsorbent with an improved AR73 removal performance in polluted aqueous and soil environments. The effects of various conditions were considered, including the adsorption time, pH, and initial mass concentration, adsorption kinetics, and thermodynamics. This study should provide a theoretical basis for alleviating aqueous and soil environments polluted by azo dyes.

## Materials and methods

### Materials

Analytical reagent (AR)-grade chitosan (CH, > 85% deacetylation) was obtained from Macklin Chemical Reagent Co., Ltd. Multiwalled carbon nanotubes (MWCNTs) with an inner diameter of 20–30 nm were purchased from Beijing Boyu Hi-Tech New Materials Co., Ltd. AR-grade glutaraldehyde, polyvinyl alcohol (PVA), glacial acetic acid, and AR73 were obtained from Sinopharm Chemical Reagent Co., Ltd. AR-grade glycerin was purchased from Yantai Aladdin Chemical Co., Ltd.

### Preparation of the composite adsorbent

**Fig. 1:**
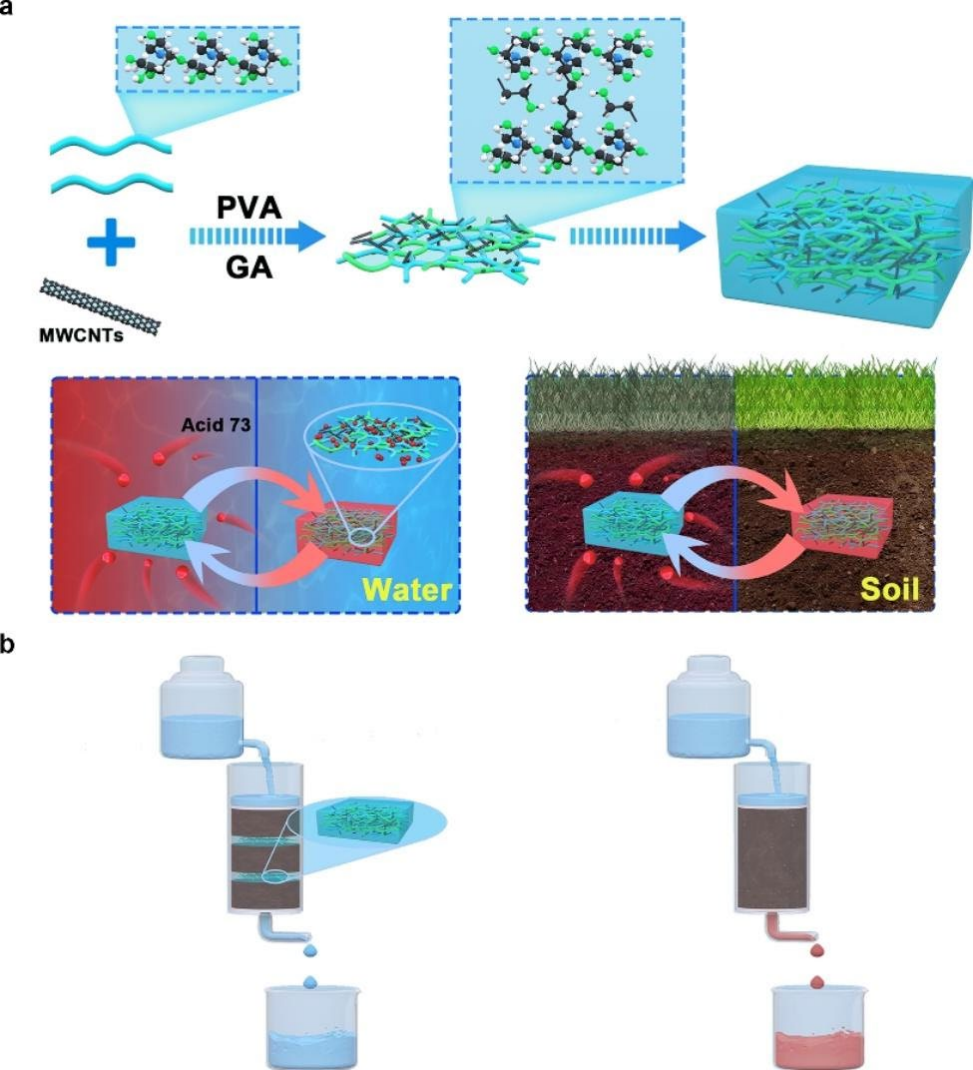
(**a**) Flowchart for preparation of the composite adsorbent (MWCNTs/CH). (**b**) Schematic of the experiment on treating AR73-contaminated soil

Figure 1(a) shows the preparation process of the composite adsorbent. First, 1 g of PVA was dissolved in 20 mL deionized water at 80 °C, and MWCNTs (0.3–1.5% by CH and PVA mass) was added for ultrasonic dispersion. After the complete dissolution of PVA in the solution, 0.25 g of CH, 0.35 mL of glacial acetic acid, 2% glutaraldehyde solution, glycerol, and deionized water were added and stirred for 40 min at 50 °C. The final solution was poured into a Petri dish and dried at 70 °C for 2 h to form a film, which was later remolded directly from the evaporating dish to obtain the composite adsorbent (MWCNTs/CH).

### Characterization of the composite adsorbent

Fourier transform infrared spectroscopy (FTIR) recorded between 500 and 4000 cm^− 1^ was performed to identify the functional groups of MWCNTs/CH using a spectrophotometer (Thermal Fisher Nicolet iS50). The surface morphology of the samples was characterized via scanning electron microscopy (SEM) (Zeiss Sigma300 and TESCAN MIRA LMS). The samples were sprayed with gold (30 mA, 90 s) before testing. The X-ray diffraction (XRD) was analysed by the Siemens D500 device with Cu-Kα (λ = 1.54056 Å)in a 2θ range of 10–80°. The BET was tested by the Siemens Micromeritics Tristar II 3020. The mechanical properties were characterized using an electronic universal testing machine (CMT6103), and the samples were prepared by following the ASTM standard. The hydrogel was cut into dumbbell samples, and the stretching rate was set to 10 mm/min. Five experiments were conducted for each sample, and the average value was obtained. An ultraviolet–visible spectrophotometer (UV-1800, Shimadzu) was utilized to measure the absorbance, which in turn could be used to calculate the adsorption capacity and AR73 removal rate (λ_max_ = 510 nm).

### Water adsorption

CH films containing mass fractions of 0%, 0.3%, 0.5%, 0.7%, and 1% MWCNTs were cut, dried completely, and weighed. MWCNTs/CH films were immersed in water for 24 h to observe their adsorption performance. The samples were weighed, and the water adsorption was calculated as follows:

1 $$r=\left(M-m\right)/m\times 100\%$$

where *M* is the mass of the MWCNTs/CH after water adsorption (g), *m* is the mass before water adsorption (g), and *r* is the water adsorption rate.

### Adsorption of acid red 73 in aqueous solution

Varying amounts of MWCNTs/CH (30–80 mg) were added to a conical flask, and 50 mL of a solution with varying concentrations of AR73 (20–300 mg/L) was removed for oscillatory adsorption. Once the adsorption was complete, the supernatant was taken. The corresponding absorbance values were measured using the ultraviolet spectrophotometer (λ_Max_ = 510 nm), and the removal rate and adsorption capacity were calculated as follows:

2 $${q}_{e}=({C}_{0}-{C}_{e})\times V/M,$$

3 $$R=1-({C}_{0}/{C}_{e})\times 100\% ,$$

where *C*_0_ and *C*_e_ are the AR73 concentrations before and after adsorption (mg/L), *V* is the adsorbate volume (mL), *M* is the adsorbent mass (g), and *q*_e_ is the equilibrium adsorption capacity of the adsorbent (mg/g). The effects of varying the amount of MWCNTs/CH, initial concentration of the solution, adsorption time, temperature, oscillation rate, and pH were considered.

### Performance of the composite adsorbent in acid red 73–contaminated soil

Contaminated soil was prepared as follows. First, 20-g soil was balanced for 1 day after being injected with 30 mL of AR73 solution. The contaminated soil was placed in a container, and MWCNTs/CH was buried in the center of the soil. Deionized water was dropped into the soil using a constant pressure funnel. The soil without MWCNTs/CH served as a control sample, and the effluent solution absorbance was measured 18 h later. Each sample group was tested thrice, and the average value was calculated. Figure 1(b) shows the experimental procedure.

### Equilibrium adsorption

AR73 solutions were produced with gradient concentrations of 2, 4, 6, 8, 10, 15, and 20 mg/L (adsorption temperature = 298 K; the adsorption time = 24 h). The Langmuir, Freundlich, and Temkin adsorption isotherms were fitted to the adsorption capacity at 298 K.

4 $$\text{Langmuir\, linear\, equation:} {C}_{e}/{q}_{e}={C}_{e}/{q}_{m}+1/b\cdot {q}_{m}$$

5 $$\text{Freundlich\, linear\, equation:} Ln{q}_{e}=Ln{K}_{f}+(1/n)Ln{C}_{e}$$

6 $$\text{Temkin\, linear\, equation:} {q}_{e}=A+BLn{C}_{e}$$

This model represents the chemical adsorption of monolayers, where *C*_*e*_ is the equilibrium adsorption concentration of the dye (mg/L), *q*_*e*_ is the adsorption capacity of the adsorbent approaching equilibrium (mg/g), *q*_*m*_ is the potential maximum adsorption capacity (mg/g), *b* is the Langmuir adsorption constant, *K*_*f*_ and *n* are the Freundlich constants, and *A* and *B* are constants.

### Adsorption kinetics

The adsorption amounts at different time were calculated at 298 K. Three equations of adsorption kinetics are given below:

7 $$\eqalign{&\text{Pseudo\, first-order\, adsorption\, kinetics:} \cr & \text{L}\text{n}({\text{q}}_{\text{e}}-{\text{q}}_{\text{t}})=\text{L}\text{n}{\text{q}}_{\text{e}}-{\text{k}}_{1}\text{t}}$$

8 $$\eqalign{&\text{Pseudo\, second-order\, adsorption\, kinetics:} \cr & t/{q}_{t}=1/\left({k}_{2}{{q}_{e}}^{2}\right)+t/{q}_{e}}$$

9 $$\eqalign{& \text{Internal\, diffusion\, equation\, of\, particles:} \cr & {\text{q}}_{\text{t}}={\text{k}}_{\text{p}}{\text{t}}^{1/2}+\text{C}}$$

where *q*_t_ represents the adsorption capacity of the adsorbent at time *t* (mg/g), *q*_*e*_ represents the adsorption capacity as adsorption equilibrium is approached (mg/g), *C* represents a parameter for the internal diffusion equation (mg/g), and *k*_1_, *k*_2_, and *k*_*p*_ represent the rate constants.

### Cyclic adsorption

Composite adsorbents the comprising adsorbed pollutants and 50 mL of 0.1 mol/L NaOH solution were placed in a conical flask, which was placed on a constant-temperature shaker. The adsorbent was removed to wash the residual NaOH solution at 100 RPM and 25 °C for 2 h. The surface water was wiped off, and the primary desorption was recorded. This process was repeated six times to observe the adsorption capacity after each cycle.

## Results and discussion

### Characterization of the composite adsorbent

#### Water adsorption

**Fig. 2:**
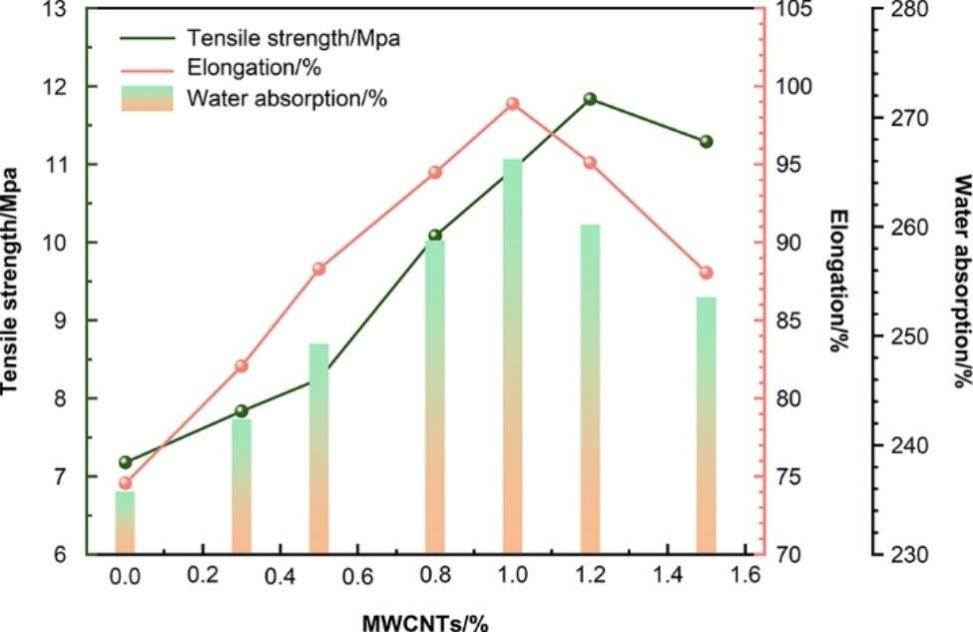
Effect of MWCNT dosage on water absorption and mechanical properties and schematic of the experiment on treating AR73-contaminated soil

Figure 2 presents the water adsorption of the MWCNTs/CH films. According to the results, the water adsorption of the film with 0 wt% MWCNTs was 235.64%, while the water adsorption of the film with 0.3 wt% MWCNTs was 242.27%. Increasing the MWCNT mass fraction caused the water adsorption of the composite adsorbent to initially increase and then decrease. The film with 1 wt% MWCNTs reached a maximum water adsorption of 266.07% owing to the high specific surface area of the MWCNTs[44]. However, further increasing the MWCNT mass fraction decreased the water adsorption because the MWCNTs agglomerated. This is consistent with the observations of the dispersion.

#### Mechanical properties

Figure 2 also shows that the mass fraction of the MWCNTs affected the tensile strength and elongation at break of the MWCNTs/CH. Compared with pure CH, adding MWCNTs improved the water absorption and mechanical both the aforementioned properties. At 0.3 wt% CNTs, the tensile strength was 7.83 MPa and the elongation at break was 82.06%. At 1 wt% CNTs, the elongation at break reached a maximum of 98.87%; compared with the elongation at break of pure CH (74.57%), this is an increase of 32.59%. At 1.2 wt% MWCNTs, the maximum tensile strength of 11.84 MPa was achieved, which is an increase of 64.90% over that of pure CH (7.18 MPa). Further increasing the mass fraction of MWCNTs degraded the mechanical properties of the composite due to the MWCNT agglomeration. This was consistent with the observations of the dispersion. Therefore, further experiments used samples with a mass fraction of 1 wt% MWCNTs [45, 46].

#### Characterizations of the structure of adsorbents

**Fig. 3:**
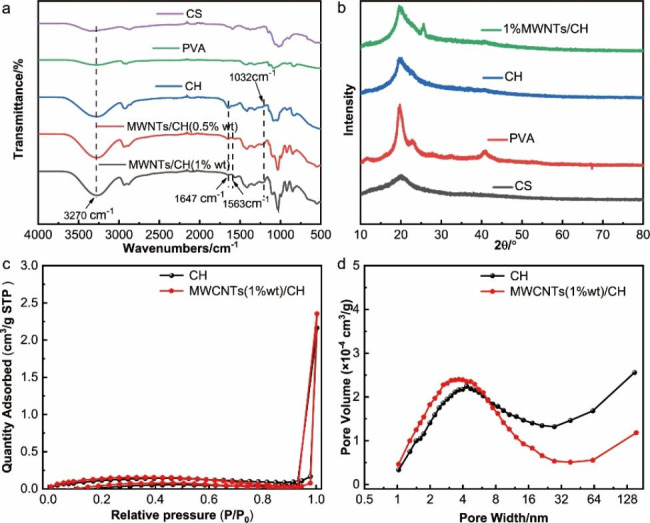
(**a**)Infrared spectra of CS, PVA, CH and MWCNTs (0.5%, 1%wt)/CH, (**b**) XRD diffractograms of CS, PVA, CH and MWCNTs (1%wt)/CH;BET analysis for as-prepared hydrogel CH and MWCNTs (1%wt)/CH samples: (**c**) N_2_ adsorption-desorption isotherm and (**d**) pore size distributions

Figure 3(a) shows the infrared spectra of CS, PVA, CH, and MWCNTs (0.5%, 1%wt)/CH. The characteristic peak at 3270 cm^− 1^ represents the stretching vibration of O–H in the CH skeleton [47]. In contrast with CS, CH and MWCNTs/CH showed strong adsorption peaks here, which indicates hydrogen bonding. The characteristic peak near 1647 cm^− 1^ is the adsorption band of the Schiff base with a C = N structure, which is formed by the reaction of the amino and aldehyde groups on the CS chain and glutaraldehyde, respectively. This proves the crosslinking between CH and glutaraldehyde. The C–O–C bond is characterized at the peak of 1120 cm^− 1^, which supports the crosslinking effect of CH. The characteristic peak near 1563 cm^− 1^ represents the C = C bond of MWCNTs in the MWCNTs/CH skeleton [19, 48].

Figure 3(b) shows the XRD of CS, PVA, CH, and MWCNTs (1%wt)/CH. X-ray diffraction (XRD) results revealed that CS was a low crystallinity polymer, as evidenced by a prominent diffraction peak observed at 2θ = 19.9°, corresponding to the (040) crystalline phases of CS. In contrast, PVA exhibited a crystallinity. Upon examining the figure, it was apparent that in the CH and MWCNTs/CH, the intensity of the diffraction peak decreased, and the peak width broadened at 2θ = 19.6°. Furthermore, other diffraction peaks associated with CS and PVA significantly diminished or even vanished. These findings suggested a robust interaction between the components and the formation of a new network structure facilitated by a chemical cross-linking reaction.Simultaneously, the presence of MWNTs in the hydrogel was confirmed by the appearance of a diffraction peak at 2θ = 25.6°, corresponding to the characteristic peak of the (002) crystalline phases of MWCNTs [19].

The specific surface area and pore size distribution of samples CH and MWCNTs (1% wt)/CH were characterized by physical adsorption desorption instrument. As shown in Fig. 3c, the adsorption capacity of the sample increases rapidly at a relative high pressure (P/P_0_ > 0.9) with no adsorption limit, and a clear H3 hysteresis loop is observed. This indicated the existence of an amount of non-uniform macropores and mesopores in samples. Furthermore, Fig. 3d presented that the pore size distribution range of CH sample is concentrated in the range of 1-155 nm. When CNTs were added in the hydrogel, the micropore volume and narrow mesoporous (2–6 nm) volume of MWCNTs (1%wt)/CH sample increased, while the pore volume with pore size greater than 27 nm decreased corresponding. In general, porosity and surface chemical properties were regarded as the key factors affecting the adsorption performance of adsorbent [49]. The volume of micropore and the specific surface area control the adsorption of small molecule pollutants [50, 51]. This indicated that CNTs in the reaction system contributed to the formation of small pore structure on the surface of the hydrogel, thus enhancing the adsorption performance of MWCNTs (1% wt)/CH.

#### Scanning electron microscopy

**Fig. 4:**
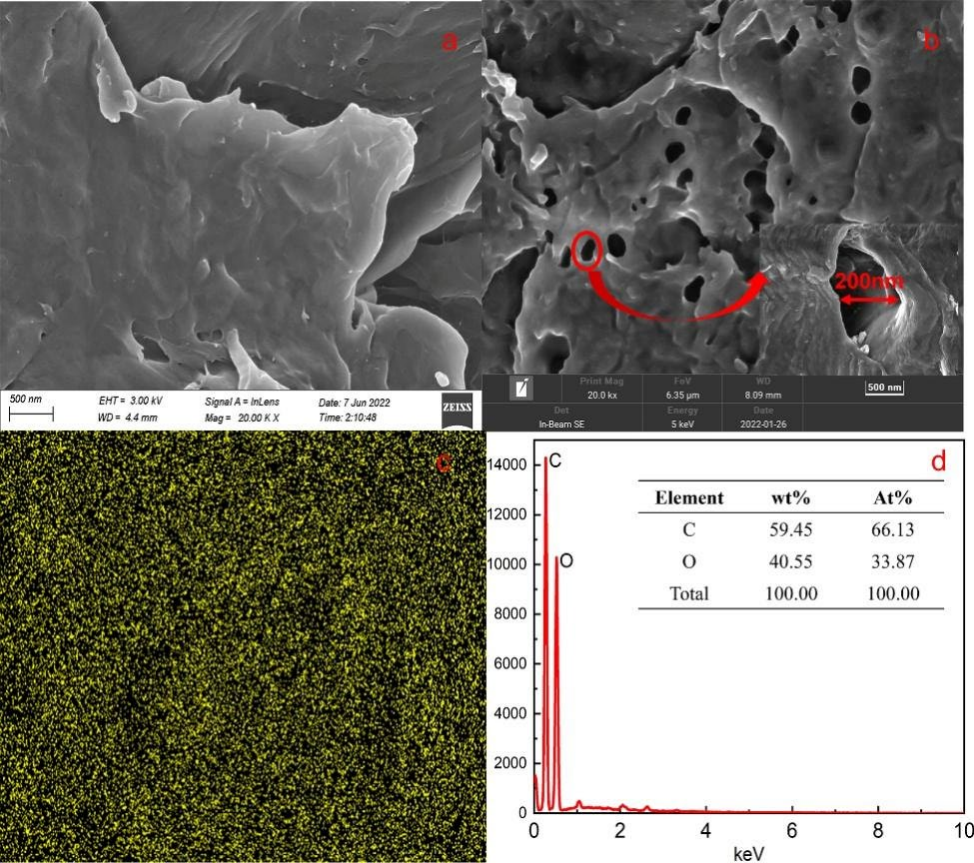
SEM morphologies of (**a**) CH and (**b**) MWCNTs/CH (1 wt%), EDX-Map analysis for MWCNTs/CH (1 wt%) (**c**) and (**d**)

The SEM images of the CH and MWCNTs/CH surfaces are illustrated in Fig. 4(a) and 4(b), respectively. The pure CH without crosslinking had a flat-sheet microstructure with no pores. For the MWCNTs/CH surface, the CH matrix showed a porous structure after crosslinking. The microstructure of MWCNTs/CH contained pores of ~ 200-nm diameter. The surface pores of MWCNTs/CH after adsorption were mostly filled with AR73. Adding MWCNTs did not damage the basic structure of the CH matrix. However, the large specific surface area of the MWCNTs effectively increased the water adsorption and AR73 adsorption performance. Simultaneously, there were no independent particles. This indicates that CH had good stability, and MWCNTs would not be separated from the film by soaking and pulling. Map-EDX analysis was alsoused to confirm the structure of MWCNTs/CH (1 wt%), that as an organic polymer hydrogel, the main element of MWCNTs/CH (1 wt%) were C and O.

### Adsorption of acid red 73 in solution and from soil

#### Effect of adsorbent mass on adsorption

**Fig. 5:**
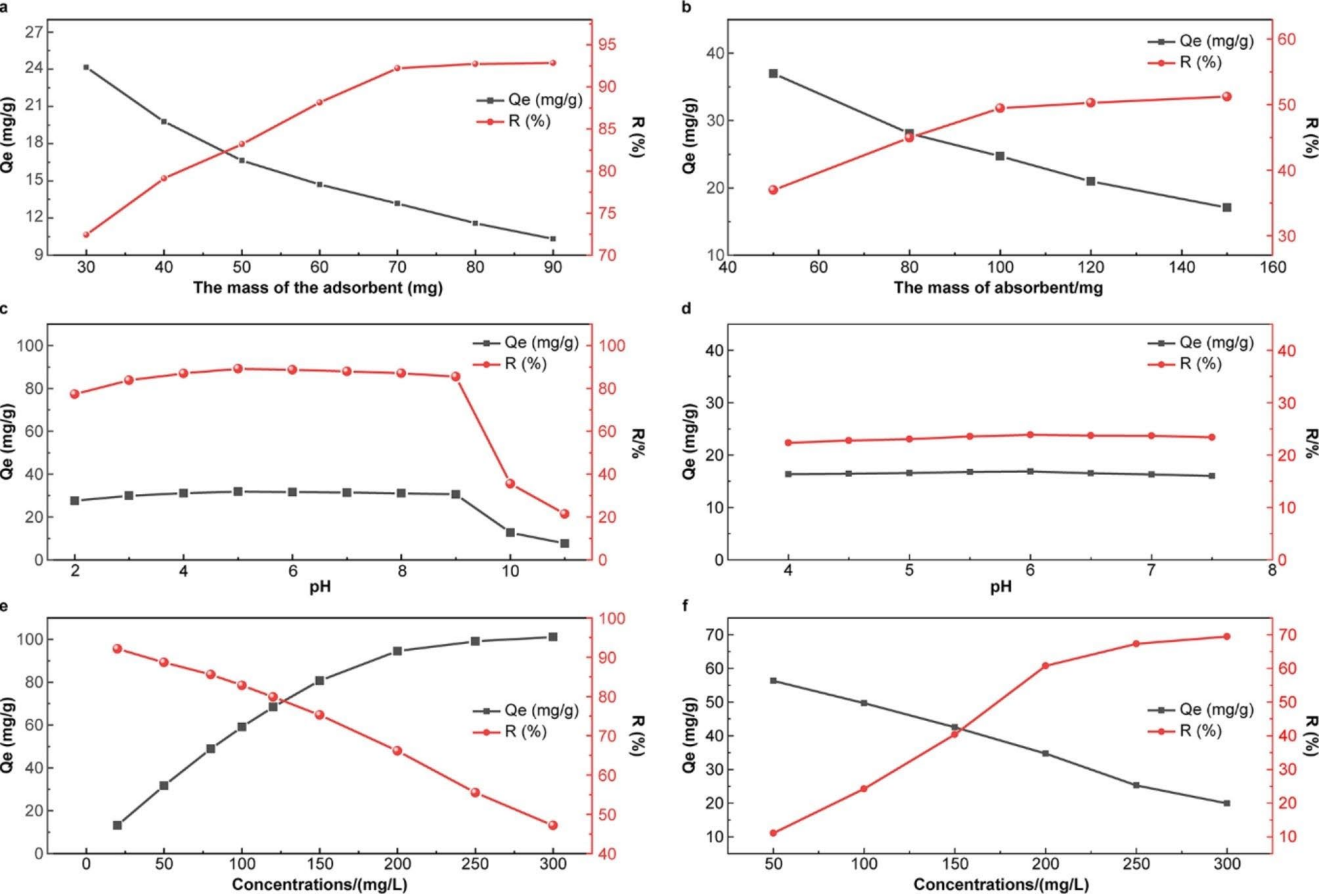
Adsorption performance: influence of adsorbent mass, pH and concentrations on AR73 adsorption in aquation (**a**), (**c**) and (**e**); and from the soil (**b**), (**d**) and (**f**)

Figure 5(a) shows the relationship between the mass of MWCNTs/CH (1.0 wt%) and the adsorption performance. The removal rate increased and then basically remain unchanged with the adsorbent mass. At 40 mg of MWCNTs/CH, the removal rate was 79.12%, whereas, at 70 mg, the maximum adsorption capacity and rate were 13.18 mg/g and 92.23%, respectively. Acid red 73 is an acidic, negatively charged dye that readily binds to –NH_2_ and –COOH groups. Chitosan is a cationic polymer; the surface of the hydrogel formed after polyvinyl alcohol modification contains a large number of –NH_2_ and –COOH groups [52]. Therefore, AR73 can be adsorbed by the chitosan hydrogel through electrostatic interactions, van der Waals forces, and hydrogen bonding (this will be discussed further in the adsorption mechanism section below). Increased surface area and adsorbent mass considerably increased the removal rate. Increasing the adsorbent mass caused the removal rate to decrease slightly. This can be attributed to the agglomeration of the adsorbent, which reduced its interaction with dye molecules. Thus, the optimal adsorbent mass for AR73 adsorption was 70 mg MWCNTs/CH.

Figure 5(b) shows how adsorbent mass affects AR73 adsorption in 20-g soil. At 50 mg of MWCNTs/CH, the AR73 was removed in 36.98% and adsorbed in 36.98 mg/g. The removal rate was proportional to the adsorbent mass. At 100 mg of MWCNTs/CH, the removal rate was 49.45%, and the adsorption capacity was 24.73 mg/g. At 150 mg of MWCNTs/CH, the removal rate was 51.23% owing to the reason in Fig. 5(a). The removal rate did not change considerably as the adsorbent mass increased. Consequently, 100 mg MWCNTs/CH were utilized in subsequent tests. The AR73 removal rate in soil samples was lower than that in aqueous solutions because chitosan hydrogels are sensitive to ions after their formation by a cationic polymer (chitosan) and nonionic hydrophilic polymer (PVA); the ions in the soil will decrease the adsorption performance. Additionally, the AR73’s poor fluidity in wet soil and competitive adsorption of other pollutants may explain this.

#### Effect of pH on adsorption

Figure 5(c) depicts the effect of solution pH on the adsorption properties in water; when the solution pH is 2–5, the removal rate and adsorption capacity increased gradually, reaching the maximum values at pH = 5 (adsorption capacity = 31.87 mg/g, and removal rate = 89.23%). Further increasing the pH to 5–9 saw a gradual to sharp decrease in removal rate and adsorption capacity. At pH = 11, the AR73 removal rate was only 21.44%. Notably, chitosan modified by polyvinyl alcohol is pH sensitive. Under acidic conditions, nitrogen-containing groups (such as –NH_2_) in hydrogels are protonated. AR73 is absorbed via electrostatic attraction, hydrogen bonding, and ion exchange. The nitrogen atom has five valence electrons; only the lone pair of electrons can interact with pollutants for adsorption. Some AR73 molecules interact with the hydrophobic groups on the hydrogel surface via van der Waals forces, which induces a dipole attraction, thereby increasing the adsorption capacity. As the pH increases, the nitrogen-containing groups on the hydrogel surface (e.g., –NH_2_) and the nitrogen and sulfur atoms in AR73 are gradually deprotonated. When the hydrogel is no longer positively charged, electrostatic repulsion to AR73 occurs, resulting in a decrease of adsorption capacity. The higher the pH value, the higher the chroma of the AR73 solution.

Figure 5(d) depicts the effect of solution pH on the adsorption performance in soil. The soil pH was typically 4–7; it can be adjusted by adding hydrated lime powder and aluminum sulfate. As can be seen from the figure, when the solution pH was 4–7, the removal rate and adsorption capacity first increased slightly with increasing pH and then decreased. The adsorption capacity and removal rate mostly remained unchanged at ~ 25.50 mg/g and 16.50%, respectively. The pH of soil suitable for plant growth is usually between 5.2 and 6.8; therefore, this hydrogel material is very suitable for treating soil pollutants [53].

#### Effect of different dye concentrations on adsorption

Figure 5(e) shows that the initial dye concentration is directly proportional to the adsorption capacity and inversely proportional to the removal rate of MWCNTs/CH in aquation. At an initial AR73 concentration of 300 mg/L, the equilibrium concentration was 171.58 mg/L, and the adsorption capacity and removal rate reached their maximum values of 101.07 mg/g and 92.11%, respectively. The adsorption capacity remained constant as the AR73 concentration was further increased, which indicates that the adsorption was saturated. This is consistent with the general flocculation effect, in which the driving force generated by the concentration gradient increases with the AR73 concentration to enhance the dye’s tendency to diffuse to the surface or internal pores of MWCNTs/CH. This increases the contact between MWCNTs/CH and AR73 and thus the adsorption of AR73 molecules to additional active sites.

Figure 5(f) shows that aquation had similar effects on the adsorption and removal rates of AR73-contaminated soil. When the dye concentration rose from 50 mg/L to 300 mg/L, the adsorption capacity increased from 11.08 to 69.46 mg/g, and the removal percentage dropped from 56.32 to 19.89%. The adsorption amount and removal rate in the aqueous solution were lower than the adsorption amount and removal rate in the soil. As the soil structure is complex and colloid-like, acid 73 molecules readily adsorb in the soil colloid rather than MWCNTs/CH. Due to the soil’s complexity, the adsorption mechanism was investigated in an aqueous solution.

**Fig. 6:**
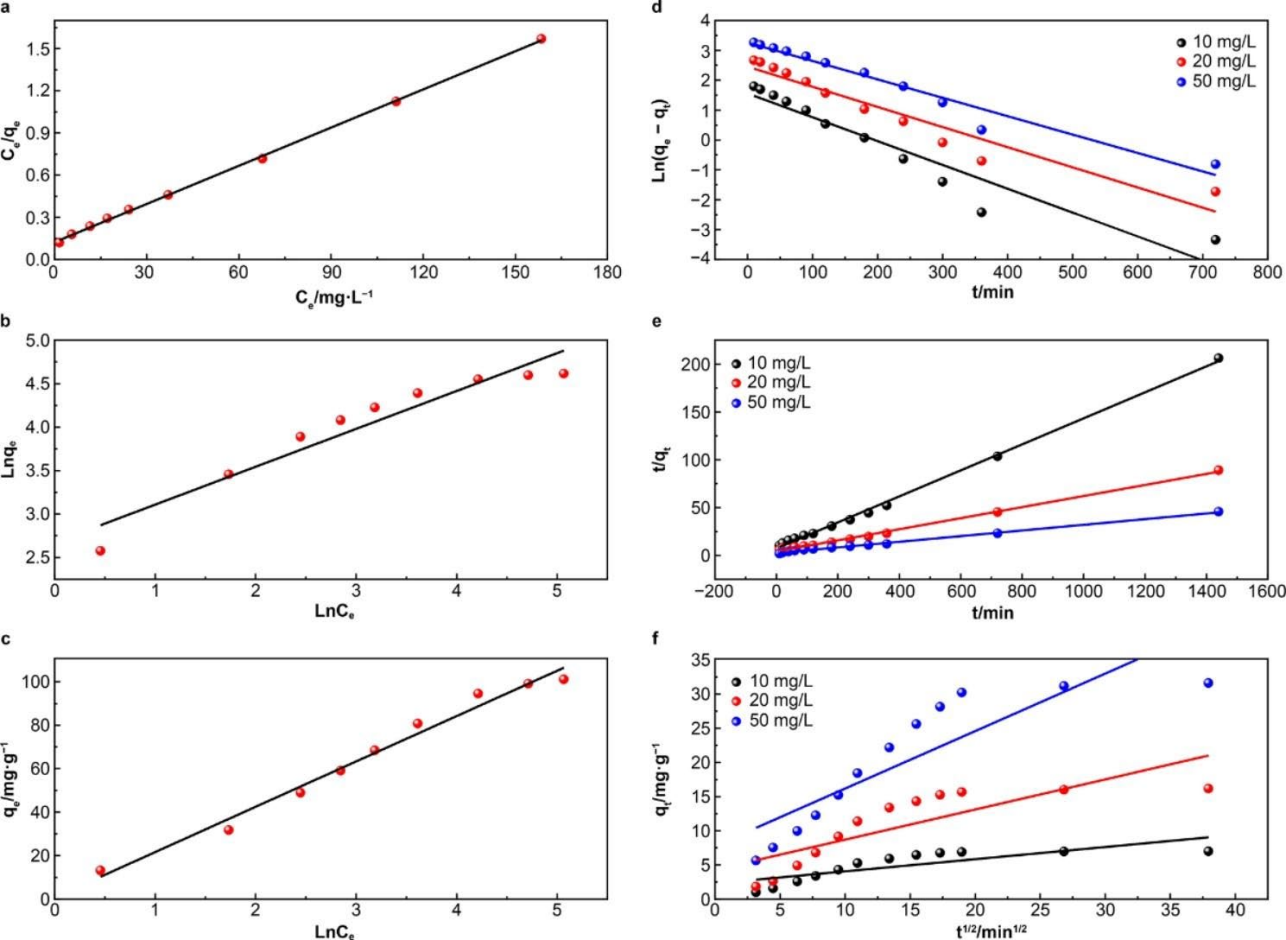
Adsorption mechanism: Isotherm fitting of AR73 adsorption on MWCNTs/CH: (**a**) Langmuir, (**b**) Freundlich, and (**c**) Temkin models and adsorption kinetics: (**d**) pseudo first-order, (**e**) pseudo second-order, and (**f**) intraparticle diffusion models

The Langmuir, Freundlich, and Temkin adsorption isotherm models were used to study the adsorption mechanism of AR73. Figure 6(a), 6(b), and 6(c) indicate that the three models fitted the test data of AR73 adsorption by MWCNTs/CH. The Langmuir model had the highest correlation coefficient (R^2^ = 0.999), which indicates that it is suitable for describing the AR73 adsorption characteristics of MWCNTs/CH. In the Langmuir model, the value of the Q_m_ is 110.50 mg/g and separation constant *R*_L_ indicates whether the adsorption behavior is irreversible (*R*_L_ = 0), favorable (0 < *R*_L_ < 1), unfavorable (*R*_L_ > 1), or linear (*R*_L_ = 1). In this study, *R*_L_ = 0.406, which indicates that the conditions were favorable for adsorption. This value also indicates that the AR73 adsorption on MWCNTs/CH is reversible and results in chemical adsorption of a monolayer [54, 55]. In the Freundlich model, 1/*n* (1/*n* = 0.436) had a value between 0.1 and 0.5, which suggests good AR73 adsorption by MWCNTs/CH.

Another anionic azo dye, Congo red, was selected for the investigation of selectivity. It was suggested that when utilizing a 50 mg MWCNTs/CH adsorbent, the adsorption capacity reached 14.16 mg/g with an adsorption rate of 88.47%. However, increasing the mass of the adsorbent resulted in a higher adsorption rate but a decrease in adsorption capacity, as shown in the supporting information (SI Fig. 1). This observation can be attributed to the presence of an -OH group within the structure of acid red 73, which readily interacts with MWCNTs/CH, forming hydrogen bonds. Consequently, this material exhibits superior adsorption efficacy towards acid red 73 [56]. In addition, the adsorption capacity of other materials to acid red 73 and similar dyes was formed in Table 1, which indicared that the MWCNTs/CH had a good adsorption properties.

**Table 1:** Adsorption capacities of various adsorbents for acid red 73 and similar dyes

adsorbents	Dye	Q_m_ (mg/g )	Source of information
Rice wine lees	Acid red 73	18.74	[20]
Chitosan hydrogel	Acid red 73	6.56	[30]
Nano-Fe_3_O_4_	Acid red 73	12.31	[31]
Lignin-based hydrogel	Acid red 73	47.59	[32]
Diethylenetriamine	Acid red 18	6.69	[57]
Sodium dodecyl sulphate	Congo red	271.74	[56]

#### Effect of time on adsorption

**Fig. 7:**
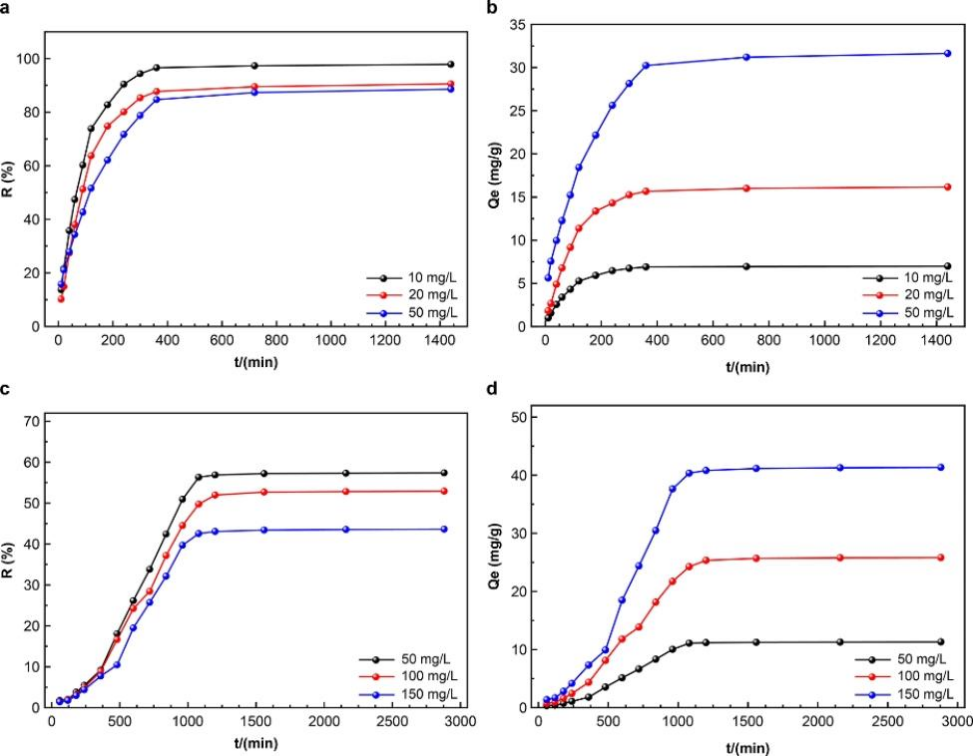
(**a**) and (**b**) influence of adsorption time on AR73 adsorption in aquation, (**c**) and (**d**) effect of adsorption time on AR73 from the soil

Figure 7(a) and 7(b) show that the AR73 adsorption process of MWCNTs/CH can be divided as fast adsorption, slow adsorption, and plateau regions. At 50 mg/L AR73, MWCNTs/CH reached an adsorption capacity of 31.64 mg/g, and the removal rate was 88.58%. Initially, the adsorption capacity increased rapidly between 0 and 1 h with a large slope. The adsorption capacity increased slowly at 1–6 h, but the adsorption efficiency increased rapidly. In the slow adsorption stage, the removal rate decreased at 6–12 h, after which the adsorption basically reached equilibrium in the plateau stage. This is due to AR73 colonizing the outer parts of the adsorbent during the preliminary phase of rapid adsorption. Over time, the surface sites were all occupied, and AR73 could only occupy the internal adsorption sites, leading to a gradual increase in adsorption capacity during the slow adsorption stage. The AR73 adsorption on MWCNTs/CH took 6 h to reach equilibrium at this temperature [58].

The impact of time on the adsorption of AR73 in soil is depicted in Fig. 7(c) and 7(d). At 150 mg/L AR73, the removal rate increased from 1.43 to 7.73% in 6 h. Wet soil and slow dye migration caused minor changes in the removal rate. After 6 h, the soil was saturated with water, and the AR73 removal rate increased from 8.90% to a maximum of 42.55% at 18 h; the adsorption capacity reached 41.36 mg/g. However, the AR73 removal rate did not increase greatly over time. The time it took for AR73 adsorption in the soil to reach parity was greater than in an aqueous solution because of the ambiguity of the soil system and other elements that affect dye migration. With sufficient adsorption time, MWCNTs/CH maintained a good AR73 removal rate in soil.

Due to the complexity of the soil, the relevant adsorption data from the solution were used for adsorption kinetics analysis. Figure 6(d), 6(e), and 6(f) and Table 2 present the relevant kinetic model and fitting data. The AR73 adsorption by MWCNTs/CH achieved equilibrium within 6 h, and the pseudo second-order adsorption kinetic equation showed good regression results with a linear correlation coefficient of > 0.99, which was superior to that of other adsorption kinetic equations. This indicates that the pseudo second-order adsorption kinetic equation accurately described the dye adsorption process. Additionally, the equilibrium adsorption capacity (*q*_*e*_, cal) calculated by the pseudo second-order adsorption kinetic equation was very close to the equilibrium adsorption capacity (*q*_e_, cal) in the experiment. This further supports the pseudo second-order adsorption kinetic equation for describing the AR73 adsorption process with MWCNTs/CH. It also indicates that the dye adsorption process is controlled by chemical adsorption. The intraparticle diffusion model of AR73 adsorption on MWCNTs/CH is provided in the supplementary files.

**Table 2:** Kinetic model parameters of AR73 adsorption on MWCNTs/CH

Concentration [mg·L^− 1^]	Q_e_(exp) [mg·g^− 1^]	Pseudo first-order			Pseudo second-order		
		Q_e_(cal) [mg·g^− 1^]	K_1_ [min^− 1^]	R^2^	Q_e_(cal) [mg·g^− 1^]	K_2_ [g·mg^− 1^·min^− 1^]	R^2^
10	6.987	4.744	0.00798	0.903	7.352	0.00254	0.997
20	16.164	11.638	0.00674	0.917	17.280	0.00079	0.995
50	31.637	26.034	0.00616	0.955	33.795	0.00037	0.996

#### Effect of temperature on adsorption

The effect of temperature on soil adsorption cannot be evaluated; hence, so only aqueous solution temperatures are considered. According to the effect of temperature on the AR73 removal rate by MWCNTs/CH, increasing the temperature slightly increased the AR73 removal rate to a maximum of 91.20% at 318 K. This may be because increasing the temperatures not only increased the mobility of the dye molecules but also caused the internal MWCNTs/CH structure to swell. Additionally, higher temperatures can make large dye molecules penetrate farther.

The adsorption free energy change (*∆G*) was calculated at different temperatures (*T*), and the linear regression method was used to fit the *T* and *∆G* values. *∆H* and *∆S* were obtained from the intercept and slope, respectively, of the equation. At the three temperatures, the thermodynamic parameters for AR73 adsorption on MWCNTs/CH were calculated and the results are presented in the supplementary files. The *∆G* values of the adsorbate were negative, which indicates the spontaneity of the AR73 adsorption on MWCNTs/CH. With increasing temperature, *∆G* and the adsorption capacity *q*_*e*_ decreased gradually, which indicated a reduction in AR73 adsorption. The values of *∆H* and *∆S* were > 0, which indicates that the AR73 adsorption by MWCNTs/CH is a spontaneous endothermic reaction that increases entropy. Particularly, the adsorption reaction occurs spontaneously at high temperature [54].

#### Cyclic adsorption

**Fig. 8:**
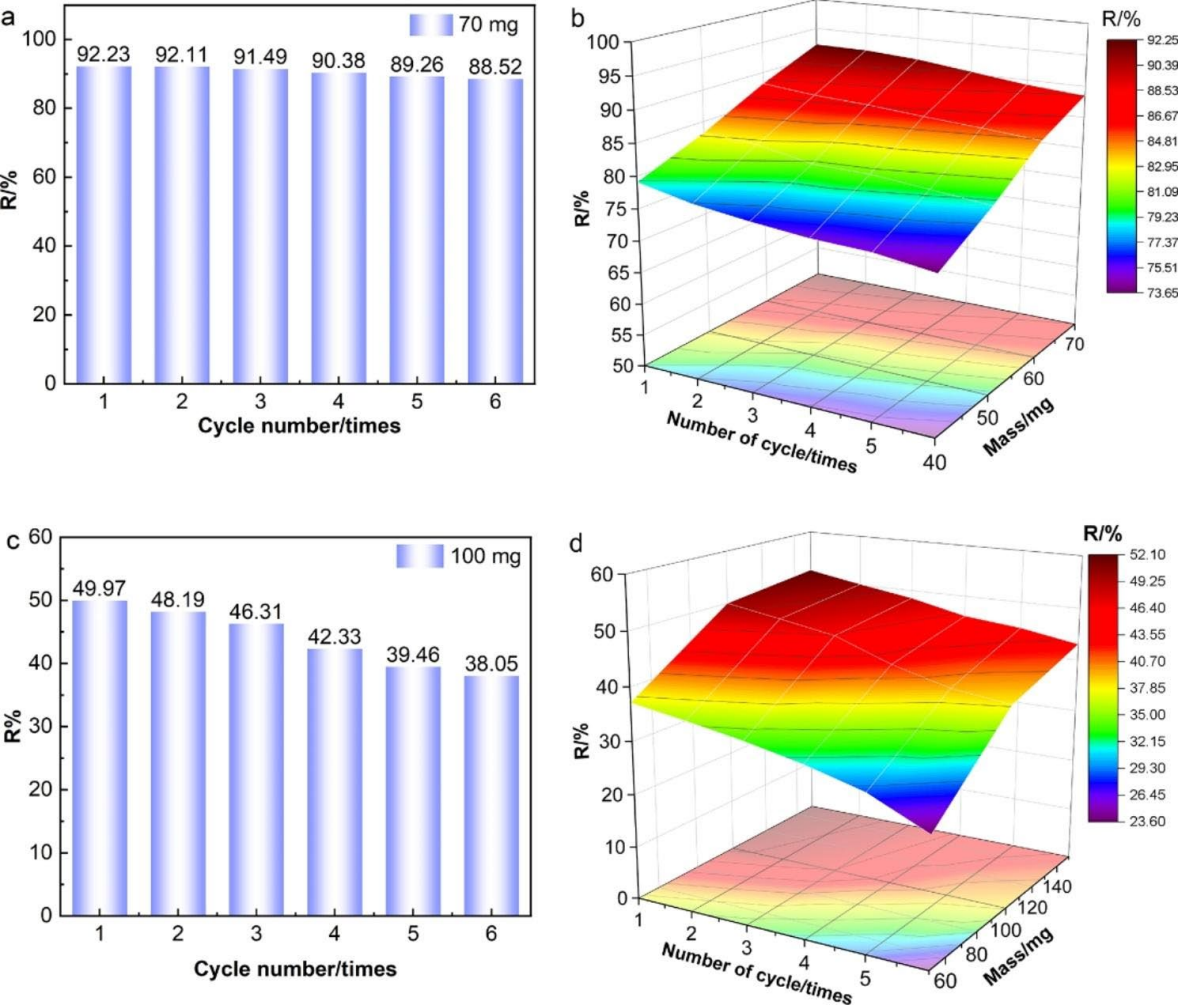
Removal rate over multiple adsorption cycles in aquation (**a**) 70 mg and (**b**) different masses of MWCNTs/CH and from the soil, (**c**) 100 mg and (**d**) different masses of MWCNTs/CH

Figure 8(a) and 8(b) show the removal rate of the cyclic adsorption of AR73 in an aqueous solution. At 70 mg of MWCNTs/CH, the removal rate of the first adsorption cycle was 92.23%. After desorption, repeated adsorption and desorption tests were performed. The removal rate remained above 90% (at 90.38%) after the fourth adsorption cycle. This is a decrease of only 1.85% compared with the initial adsorption cycle, which indicates that MWCNTs/CH retained excellent adsorption capacity. At six adsorption cycles, the AR73 removal rate from the aqueous solution was 88.52%, which was a 3.71% decrease from the initial adsorption cycle but was still high. At 40 mg of MWCNTs/CH, after six adsorption cycles, AR73 removal rate decreased from initial 79.12–73.68%, which is a decrease of 5.44%. At 50 mg of MWCNTs/CH, six adsorption cycles decreased the AR73 removal rate from 83.20 to 78.75%, which is a decrease of 4.45%. At 60 mg MWCNTs/CH, six adsorption cycles decreased the AR73 removal rate from 88.15 to 84.69%, which is a decrease of 3.46%. Regardless of the initial adsorbent mass, the AR73 removal rate remained relatively high throughout the adsorption cycles, although it did decrease after six cycles due to decreases in the specific surface area and functional group strength. This can be attributed to the gel strength of the hydrogel. When coupled with the three-dimensional network structure, the composite adsorbent can store large amounts of water in its own structure to allow a greater degree of reuse. Thus, MWCNTs/CH showed excellent recyclability in aqueous solutions. Recyclability is an important parameter for evaluating the adsorbent performance because it reduces not only reduces the cost of adsorbents but also reduces environmental pollution.

Figure 8(c) and 8(d) show the AR73 removal rate in soil over increasing adsorption cycles. Figure 8(c) shows that at 100 mg of MWCNTs/CH, the removal rate was 49.97% after the first adsorption cycle, and it was 48.19% and 46.31% after the second and third adsorption cycles, respectively. Thus, the removal rate decreased by only 3.66% over three cycles. After six adsorption cycles, the removal rate was 38.05%, which is a decrease of 11.92%. However, MWCNTs/CH maintained a good AR73 removal rate. Figure 8(d) shows that changing the adsorbent mass to 50 and 150 mg MWCNTs/CH decreased the AR73 removal rate after six cycles. At 50 mg of MWCNTs/CH, the AR73 removal rate in soil dropped from an initial 37.13–23.60%. At 150 mg of MWCNTs/CH, the AR73 removal rate decreased from initial 51.23–43.09%, which is a reduction of 8.14%. These results indicate that MWCNTs/CH is not only effectively at treating AR73-contaminated soil but also can be used repeatedly. Thus, it has great application potential and value. Moreover, after six cycles of use, the mechanical properties of the hydrogel film were tested again. The tensile strength in water decreased from 10.93 MPa to 10.77 MPa, and the tensile strength in soil decreased to 10.32 MPa. The tensile strength and elongation at break did not change considerably, thus the material has sufficient stability for repeated use.

**Fig. 9:**
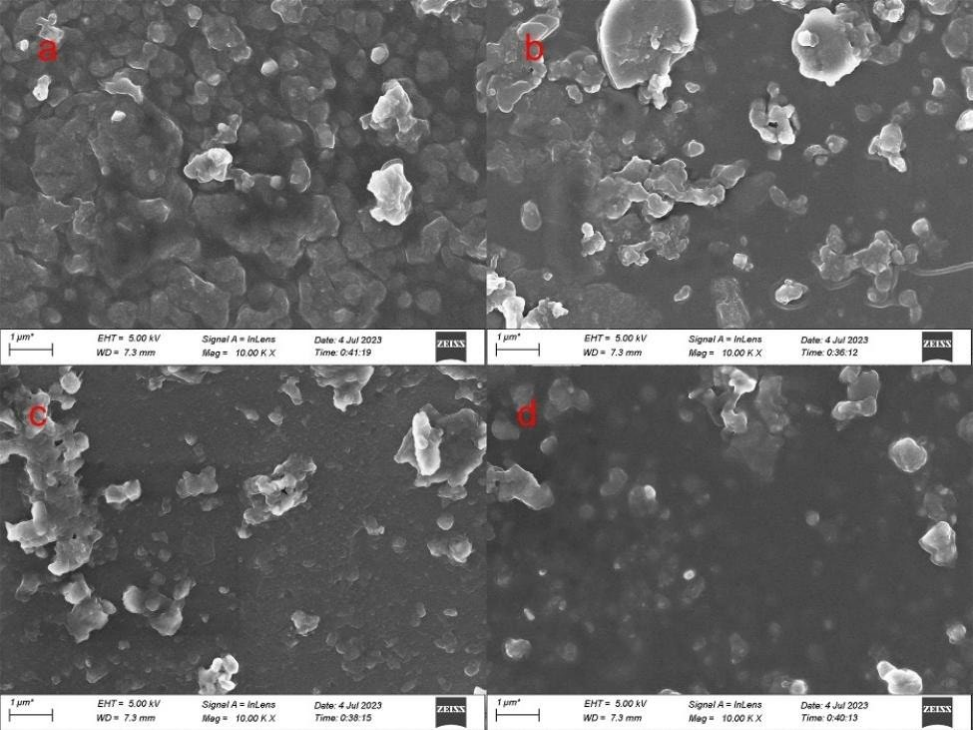
SEM morphologies of MWCNTs/CH (1 wt%) after adsoption in aquation after one (**a**) and (**b**)six times adsorption time adsorption in aquation; one (**c**) and six (**d**) times from soil

Figure 9 presented the SEM images of MWCNTs(1%wt)/CH before and after undergoing one and six cyclic adsorption processes in both aquation((a) and (b) ) and soil((c) and (d) ) environments. The figure clearly illustrated that the pores of MWCNTs(1%wt)/CH were completely filled after adsorption. Furthermore, upon comparing the morphology of the material after six cycles of adsorption with that after the initial adsorption, it was observed that there was no significant alteration, thereby indicating a high level of stability.

### The adsorption mechanism

For hydrogels, swelling and absorption capacity are the two most basic and important properties of hydrogels as well as the basic factors affecting absorbability and recyclability. Therefore, in this study, natural and pollution-less chitosan was selected as the substrate, nonionic hydrophilic polymer polyvinyl alcohol was added, glutaraldehyde was used as the crosslinking agent, and carbon nanotubes were added to the chitosan hydrogel. A chitosan interpenetrating network hydrogel film with high strength and good water absorption was formed [59].

Chitosan hydrogel molecules is pH sensitive after forming a hydrogel with nonionic hydrophilic polymers(PVA) with abundant –NH_2_. Under acidic conditions, the nitrogen-containing groups (such as –NH_2_) in the hydrogel are protonated to form –NH_3_^+^, which is a cationic polymer. Acid red 73 is an acid dye that dissociates anion sulfonate in water, which is a water-soluble sulfonate, and the dye ions are negatively charged. This can be demonstrated by the effect of the change of pH on the adsorption results described above. Additionally, as the FTIR results showed, some AR73 molecules interacted with hydrophobic groups on the surface of the hydrogel through van der Waals force, which induced dipole attraction, thereby increasing the adsorption capacity. Moreover, hydrogen bonds present between the molecules can be combined to remove pollutants.

## Conclusions

In this study, an MWCNTs/CH film with a three-dimensional network structure was prepared and the MWCNTs(1%wt)/CH with optimum properties was applied to absorb AR73 during aquation and from soil. The experimental results showed that the absorption capacity of MWCNTs/CH in water increased to a much higher value (101.07 mg/g) than that reported in the existing literature and the removal rate increased to 92.23%, that tended to follow the Langmuir model and pseudo second-order adsorption kinetic equation. Additionally, the absorption capacity of the absorbent was studied in soil; the adsorption capacity and removal rate were 24.73 mg/g and 49.45%, respectively, which has not been previously demonstrated. Meanwhile, MWCNTs/CH retained its high AR73 removal rate over multiple adsorption cycles in both the aqueous solution and soil samples, highlighting its recyclability and practical application value.

### Electronic supplementary material

Below is the link to the electronic supplementary material.

Supplementary Material 1

## Data Availability

Data available on request from the authors.
