# Supplementary material for: Carbon nanotube/Chitosan hydrogel for adsorption of acid red 73 in aqueous and soil environments
Source: BMC Chem. 2023 Aug 24;17(1):104. doi: 10.1186/s13065-023-01019-9 (PMC10463536; doi:10.1186/s13065-023-01019-9)
Supplement: Supplementary file 1 — Supplementary Material 1 [file 13065_2023_1019_MOESM1_ESM.docx]

1. Selectivity of MWNTs(1%wt)/CH with Congo red





Fig 1 Influence of adsorbent mass on Congo red adsorption in aquation

It was suggested that when utilizing a 50 mg MWCNTs/CH adsorbent, the adsorption capacity reached 14.16 mg/g with an adsorption rate of 88.47%. However, increasing the mass of the adsorbent resulted in a higher adsorption rate but a decrease in adsorption capacity.

[1]N.A. Travlou, G.Z. Kyzas, N.K. Lazaridis, E.A. Deliyanni, Functionalization of graphite oxide with magnetic chitosan for the preparation of a nanocomposite dye adsorbent, Langmuir 29 (2013) 1657–1668
